# On Two Cases of Poisoning by Anilides (Exalgine and Antifebrin)*Read in the section of Pharmacology and Therapeutics at the annual meeting of the British Medical Association, held in Leeds.

**Published:** 1890-03

**Authors:** T. Jessopp Bokenham, E. Lloyd Jones


### ON TWO CASES OF POISONING BY ANILIDES (EXALGINE AND ANTIFEBRIN).*

BY T. JESSOPP BOKENHAM, M. R. C. S., L. R. C. P., L. S. A.,
AND
E. LLOYD JONES, M. B. B. C. (CAMB.)

Considering the large number of artificial drugs which have recently come into general use, and the imperfect state of our knowledge as regards their exact physiological action, it is somewhat surprising that more mishaps have not occurred as a consequence of their use. Although acetanilide (antifebrin), antipyrin, and similar drugs are very largely prescribed, and are even used by patients on their own responsibility, yet their seems, judging from the English journals, to have been a considerable immunity from alarming toxic effects. This being the case, we feel that no apology is needed for recording the two following cases.

CASE I.—M. L, a girl, aged 24 years, had myelitis, with great pain in the back and limbs. There was no elevation of temperature. On June 3d it was decided to give exalgin (methylacetanilide) with a view of relieving the pain. From this date until June 8th the drug was given in doses of gr. ij ter die. The dose was then doubled, but as even this amount failed to give relief it was further increased on June 10th to gr. vj ter die, and continued until June 17th, when symptoms of poisoning first set in. At 10:30 A. M. on the 17th the lips and cheeks were noticed to be blue, the pulse was small and compressible, but not rapid, temperature being normal. Though there were no very urgent symptoms the drug was discontinued, and brandy ʒij was ordered every hour. The patient stated that the medicine made her feel "sick and giddy," that her sight was indistinct, and that there was a feeling of weight at the epigastrium. At 3:30 P. M. a few inhalations of amyl nitrite were given; this considerably empha-

*Read in the section of Pharmacology and Therapeutics at the annual meeting of the British Medical Association, held in Leeds.

sized the blueness, and caused marked dilatation of the vessels. It was thus evident that the whole of the circulating blood was profoundly changed. The cyanosis continued to increase, and at 3:45 P. M. the patient vomited, after which it became still more marked. At 4:10 P. M. her nails, lips, and cheeks were deeply cynosed; frothy saliva was escaping from her mouth; she was delirous, and appeared to recognize no one. Her feet were not blue nor cold. Temperature 99.8° F.; pulse 144, very small, regular, compressible. The amount of stimulant was increased, and liq. strychnine *m*ij given hypodermically, a mixture containing tinct. digitalis *m*x being also given by the mouth. She began to improve, and at 9 P. M. her condition, save for slight cyanosis, was fairly normal. She slept well at night, and had no further untoward symptom.

As regards this patient's previous condition it should be stated that her bowels had been opened only by purgatives and had not acted on June 14th, 15th or 16th. She had not menstruated for some months.

CASE II.—K. B., a lady, aged 43, very subject to migraine, was seized with a severe attack shortly after rising on June 5th. She had been for some months much relieved by antipyrin on these occasions, but latterly this had failed; acetanilide, however, in doses of 5 grammes, had generally been successful in relieving the pain.

A short time before this date, thinking that the introduction of a bromide atom into the molecule would render acetanilide more powerful as an analgesic, one of us had obtained a specimen of monobromacetanilide from Schuchardt, of Gorlitz. Having used this body with personal benefit, the patient was given two powders (0.25 gramme in each) to use for the next migraine. At 10:30 A. M. the patient took one of these powders, and, getting no relief, at 11 A. M. took a second, At about this time she noticed her lips were rather blue, but laid no stress on it. Finding the headache no better, at 11:30 she, of her own accord, took 0.5 gramme of acetanilide, and repeated this dose at 12:15 and 1 P. M.; headache not relieved. She had a good appetite for lunch, but it was noticed that she was very cyanosed, and she confessed to feeling "almost intoxicated, and very giddy." She took some whisky and lay down, but the headache became worse. At about 3 P. M. her clothes "felt tight, and the pressure almost suffocating." She tried to undress, but was seized with pain in the left shoulder starting from the cardiac region, and extending to the tips of the fingers. She was quite unable to call any one to help her, and lost consciousness. When found soon after, however, she was conscious again, feeling only giddy; the headache persisted, and the pain in the arm returned with every attempt at movement.

At 5 P. M. her condition was as follows: The pulse was 108 and easily extinguished; the respiration 24, somewhat shallow; the temperature was 99° F.; heart's action feeble, and a mitral systolic murmur at apex; mental condition perfectly normal; arm still painful on movement, and a tendency to close the eyes unless speaking. On being questioned as regards this closure of the eyes, she answered that it was only because it "wasn't worth while keeping them open"—not because she was drowsy. She felt hungry and thirsty, and took some tea with bread and butter. The cyanosis was said to be less intense than in the afternoon.

At 7 P. M., the heart being very weak, nitro-glycerine $\frac{1}{100}$ grain was given. Great dilatation of vessels was produced, with increase of cyanosis. The heart sounds were feeble; there was no murmur. Alcohol, strychnine, and digitalis were now given at frequent intervals, and the cyanosis diminished.

At 11 P. M. the temperature was 100.2 degrees F.; pulse 120, soft; respirations 28. Faintness was induced by the least movement, and pain in the shoulder and arm was still present; headache no better; cyanosis much less. Next morning, at 11, the temperature was 99.4 degrees F.; the patient had not slept during the night, but the cyanosis was almost gone, the headache was better, and the pulse stronger; hands distinctly puffy. A stimulant treatment was adopted through the day, and in the evening the patient was practically well. Urine was watched for albumen and blood residues, but none was found. No very marked anæmia followed the attack. It is to be noticed, also, that this patient became ill on the third day of normal menstruation. Bowels were perfectly normal throughout.—*Brit. Med. Journal, Feb. 1, 1890.*

